# Supplementary material for: Resolving the full spectrum of human genome variation using Linked-Reads
Source: Genome Res. 2019 Apr;29(4):635–45. doi: 10.1101/gr.234443.118 (PMC6442396; doi:10.1101/gr.234443.118)
Supplement: Supplemental Material [file supp_gr.234443.118_Supplemental_longranger-2.2.zip › longranger-2.2.tar.bz2!/lr2.2-public/lib/stan/cmdstan/stan/doc/api/html/fwd_2functions_2fmax_8hpp_source.html]

Stan: src/stan/agrad/fwd/functions/fmax.hpp Source File


|  |  |
| --- | --- |
|  | Stan  2.5.0  probability, sampling & optimization |


- Main Page
- Related Pages
- Namespaces
- Classes
- Files

- File List
- File Members

All Classes Namespaces Files Functions Variables Typedefs Enumerations Enumerator Friends Macros Pages

- src
- stan
- agrad
- fwd
- functions

fmax.hpp

Go to the documentation of this file.

1 #ifndef STAN\_\_AGRAD\_\_FWD\_\_FUNCTIONS\_\_FMAX\_HPP

2 #define STAN\_\_AGRAD\_\_FWD\_\_FUNCTIONS\_\_FMAX\_HPP

3

4 #include <stan/agrad/fwd/fvar.hpp>

5 #include <stan/meta/traits.hpp>

6 #include <stan/math/constants.hpp>

7 #include <math.h>

8 #include <stan/meta/likely.hpp>

9

10 namespace stan {

11

12  namespace agrad {

13

14  template <typename T>

15  inline

16  fvar<T>

17  fmax(const fvar<T>& x1, const fvar<T>& x2) {

18  using ::fmax;

19  using stan::math::NOT\_A\_NUMBER;

20  if (unlikely(boost::math::isnan(x1.val\_))) {

21  if (boost::math::isnan(x2.val\_))

22  return fvar<T>(fmax(x1.val\_,x2.val\_),NOT\_A\_NUMBER);

23  else

24  return fvar<T>(x2.val\_, x2.d\_);

25  } else if (unlikely(boost::math::isnan(x2.val\_)))

26  return fvar<T>(x1.val\_,x1.d\_);

27  else if(x1.val\_ > x2.val\_)

28  return fvar<T>(x1.val\_, x1.d\_);

29  else if(x1.val\_ == x2.val\_)

30  return fvar<T>(x1.val\_, NOT\_A\_NUMBER);

31  else

32  return fvar<T>(x2.val\_, x2.d\_);

33  }

34

35  template <typename T>

36  inline

37  fvar<T>

38  fmax(const double x1, const fvar<T>& x2) {

39  using ::fmax;

40  using stan::math::NOT\_A\_NUMBER;

41  if (unlikely(boost::math::isnan(x1))) {

42  if (boost::math::isnan(x2.val\_))

43  return fvar<T>(fmax(x1,x2.val\_),NOT\_A\_NUMBER);

44  else

45  return fvar<T>(x2.val\_, x2.d\_);

46  } else if (unlikely(boost::math::isnan(x2.val\_)))

47  return fvar<T>(x1,0.0);

48  else if(x1 > x2.val\_)

49  return fvar<T>(x1, 0.0);

50  else if(x1 == x2.val\_)

51  return fvar<T>(x2.val\_, NOT\_A\_NUMBER);

52  else

53  return fvar<T>(x2.val\_, x2.d\_);

54  }

55

56  template <typename T>

57  inline

58  fvar<T>

59  fmax(const fvar<T>& x1, const double x2) {

60  using ::fmax;

61  using stan::math::NOT\_A\_NUMBER;

62  if (unlikely(boost::math::isnan(x1.val\_))) {

63  if (boost::math::isnan(x2))

64  return fvar<T>(fmax(x1.val\_,x2),NOT\_A\_NUMBER);

65  else

66  return fvar<T>(x2, 0.0);

67  } else if (unlikely(boost::math::isnan(x2)))

68  return fvar<T>(x1.val\_,x1.d\_);

69  else if(x1.val\_ > x2)

70  return fvar<T>(x1.val\_, x1.d\_);

71  else if(x1.val\_ == x2)

72  return fvar<T>(x1.val\_, NOT\_A\_NUMBER);

73  else

74  return fvar<T>(x2, 0.0);

75  }

76  }

77 }

78 #endif

boost::math::isnan

bool isnan(const stan::agrad::var &v)

Checks if the given number is NaN.

**Definition:** boost\_fpclassify.hpp:69

stan::agrad::fmax

var fmax(const double &a, const stan::agrad::var &b)

Returns the maximum of a scalar and variable, promoting the scalar to a variable if it is larger (C99...

**Definition:** fmax.hpp:132

stan::math::NOT\_A\_NUMBER

const double NOT\_A\_NUMBER

(Quiet) not-a-number value.

**Definition:** constants.hpp:53

stan::agrad::fvar

**Definition:** fvar.hpp:13

stan::agrad::fmax

fvar< T > fmax(const fvar< T > &x1, const fvar< T > &x2)

**Definition:** fmax.hpp:17

traits.hpp

unlikely

#define unlikely(x)

**Definition:** likely.hpp:9

fvar.hpp

likely.hpp

stan::agrad::fvar::d\_

T d\_

**Definition:** fvar.hpp:16

constants.hpp

stan::agrad::fvar::val\_

T val\_

**Definition:** fvar.hpp:15


---

[ Stan Home Page ]

*© 2011–2014,
Stan Development Team.*
